# Detecting chirality-induced spin selectivity in chromophore-linked DNA hairpins using photogenerated radical pairs

**DOI:** 10.1073/pnas.2515120122

**Published:** 2025-08-05

**Authors:** Elisabeth I. Latawiec, Alessandro Chiesa, Yunfan Qiu, Nikolai A. Tcyrulnikov, Ryan M. Young, Stefano Carretta, Matthew D. Krzyaniak, Michael R. Wasielewski

**Affiliations:** ^a^Department of Chemistry, Institute for Quantum Information Research and Engineering, and Center for Molecular Quantum Transduction, Northwestern University, Evanston, IL 60208-3113; ^b^Dipartimento di Scienze Matematiche, Fisiche e Informatiche, Università di Parma, Parma I-43124, Italy; ^c^Consorzio Interuniversitario Nazionale per la Scienza e Tecnologia dei Materiali, Unità di Ricerca, Università di Parma, Parma I-43124, Italy; ^d^Istituto Nazionale di Fisica Nucleare - Sezione Milano-Bicocca, Gruppo Collegato di Parma, Parma I-43124, Italy

**Keywords:** radical ion pair, chirality, CISS, spin dynamics, electron transfer

## Abstract

Chirality-induced spin selectivity (CISS) results in spin polarization of electrons transmitted through chiral molecules and materials. Since CISS results in spin polarization even at room temperature, it affords the possibility of using it to develop quantum technologies that can operate under ambient conditions. We have shown previously that photo-driven hole transfer within DNA hairpins provides a facile route to generate spin-correlated radical pairs (SCRPs). To study the effect of CISS on the spin dynamics of SCRPs in DNA hairpins, we prepared a series of electron donor—chiral bridge—acceptor molecules where the chiral bridge is a B-form DNA helix consisting of 4 to 6 base pairs. Naphthalene-1,8:4,5-bis(dicarboximide) (NDI) serves as the hairpin linker chromophore and electron acceptor. Photoexcitation of NDI results in rapid hole transfer through the π-stacked purine bases of the DNA and trapping of the hole on a terminal stilbene diether (Sd) to generate the NDI^•−^- Sd^•+^ SCRP. Time-resolved electron paramagnetic resonance spectra of the SCRPs at X- (9.6 GHz), Q- (34 GHz), and W- (94 GHz) bands show that the CISS effect imparts significant triplet character to the SCRP. We do not observe a significant dependence of CISS on DNA length, likely resulting from hole delocalization over the guanine bases in the G-tract. Interestingly, we find that the CISS contribution significantly increases with magnetic field strength. These findings should be considered in any future modeling of CISS.

## Significance

Chirality (or handedness) is ubiquitous in Nature. About 25 y ago, it was discovered that transmitting electrons through a chiral molecule results in electron spin polarization, a phenomenon called Chirality-Induced Spin Selectivity (CISS). The DNA helix is chiral and can serve as a way to explore CISS by monitoring the spin characteristics of photo-driven charge transfer through short DNA hairpins having a photoexcitable hairpin linker and a hole trap at the other end of the DNA duplex. Time-resolved electron paramagnetic resonance spectroscopy of the radical ion pairs produced following photoexcitation of the DNA hairpin linker shows that CISS significantly influences the spin transferred through the hairpins affording the possibility that DNA can serve to control spin states in quantum devices.

Quantum information science (QIS) has the potential to revolutionize fields such as computing (1, 2), communications (3), and sensing (4). Molecular systems are emerging as promising QIS platforms due to the power of chemical synthesis to tune quantum properties on an atomic scale (1). Recently, the prospect of utilizing chirality in molecules to manipulate quantum states has gained considerable interest. In an effect termed chirality-induced spin selectivity (CISS), electrons or holes that are transmitted through a chiral molecule or material emerge with a preferred spin state that depends on the chirality of the substance (5–10). CISS enables spin polarization even at room temperature, making it an approach to quantum technologies that may operate under more practical, ambient conditions (6).

Although many theoretical models have been proposed to explain spin polarization through CISS, a comprehensive understanding is lacking (10–16). Spin–orbit coupling (SOC) within the chiral molecule is often cited as the source of CISS. However, in organic molecules lacking significant SOC, the observed spin polarization magnitudes are much larger than those predicted (11, 12). Some models include additional factors, such as electron–electron (13) or electron–vibration interactions (14, 15), which may enhance spin polarization beyond contributions from SOC. Alternatively, other models propose that the metal surface to which the chiral molecule is attached during measurements provides the large SOC responsible for CISS (10, 16). To better understand the phenomenon and disentangle the role of the surface from the chiral bridge, it is important to examine CISS independently of the metal surface.

Following recent theoretical predictions (17–19), we demonstrated that CISS exhibits distinct signatures in the time-resolved electron paramagnetic resonance (TREPR) spectra of a singlet-born, spin-correlated radical pair (SCRP) produced by two-step, subnanosecond electron transfer in a donor-chiral bridge-acceptor (D-Bχ-A) triad following photoexcitation of the donor to yield D^•+^-Bχ-A^•−^ (20). We found that the observation of CISS is sensitive to the orientation of D^•+^-Bχ-A^•−^ relative to an externally applied magnetic field and this orientation dependence results in clear lineshape changes to the TREPR spectra. In subsequent work, we examined a different D-Bχ-A triad in which photoexcitation of the acceptor results in hole transfer through the chiral bridge followed by hole trapping on the donor (21). Once again, lineshape changes to the TREPR spectra of the D^•+^-Bχ-A^•−^ SCRP are observed that are characteristic of a significant CISS contribution to its spin dynamics. Further, the favorable magnetic parameters of the D^•+^ and A^•−^ radical ions allow CISS to be observed even when the molecules are randomly oriented in a solvent glass at cryogenic temperatures (21). These findings shift the focus toward understanding the role of the chiral bridge in contributing triplet character to SCRPs through the CISS effect (22, 23).

To gain a deeper understanding of CISS, we sought a system in which the nature of the chiral bridge can be changed rapidly with wide scope. We have shown previously that photo-driven hole transfer through the base pairs in DNA hairpins provides a facile route to SCRPs (24–28) that takes advantage of the broad base of charge transfer information that we have established for these systems (29, 30). In these hairpins, the photoexcited chromophore serves as the hairpin linker and electron acceptor, thereby launching a hole into the purine bases of the hairpin. Attaching a stilbene diether (Sd) electron donor that serves as a hole trap to the 5′ strand of the duplex results in rapid formation of an SCRP. In particular, the diblock DNA structure comprising sequential A-T and G-C base pairs forms a chiral bridge between the donor and acceptor taking advantage of the seminal observations of CISS for charge transport through DNA (31). Moreover, the well-established library of chromophore linkers and base-pair sequences make DNA hairpins a convenient platform for investigating the nature of CISS (29). For example, by varying the number of base pairs in the bridge, we can study how the contribution of CISS to the SCRP spin dynamics depends on the length of the chiral bridge. Furthermore, by varying the resonance frequency of the TREPR experiments, we can study the dependence of CISS on the applied magnetic field strength.

**Fig. 1.**
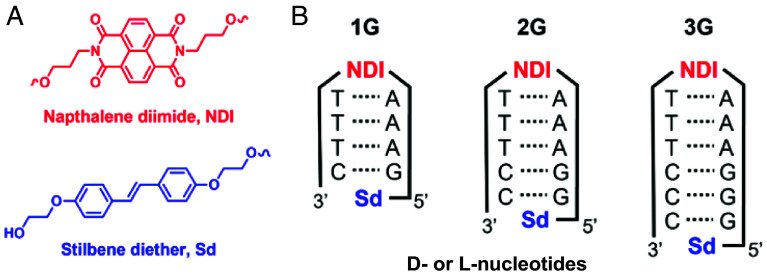
(*A*) Structures of the NDI chromophore/electron acceptor and the Sd terminal electron donor. (*B*) Hairpin structures.

Thus, we prepared a series of D-Bχ-A molecules where Bχ is a B-form DNA helix consisting of 4 to 6 base pairs (Fig. 1) and we probed the spin dynamics of the photogenerated D^•+^-Bχ-A^•−^ SCRP at different applied magnetic fields. Naphthalene-1,8:4,5-bis(dicarboximide) (NDI) serves as the hairpin linker chromophore and electron acceptor. Photoexcitation of NDI results in rapid hole transfer through the π-stacked purine bases of the DNA and trapping of the hole on the terminal Sd hole acceptor to generate the NDI^•−^-Sd^•+^ SCRP.

TREPR spectra of the SCRPs at X- (9.6 GHz), Q- (34 GHz), and W- (94 GHz) bands provide evidence that an important contribution from CISS must be included to simulate the spectra. However, we do not observe a significant dependence of CISS on DNA length, likely owing to hole delocalization over the guanine bases in the G-tract (32, 33). Conversely, we find a significant increase in the CISS contribution to the TREPR spectra with increasing magnetic field strength. These findings should be considered in any future modeling of CISS.

## Results and Discussion

### DNA Hairpin Design and Characterization.

**Fig. 2.**
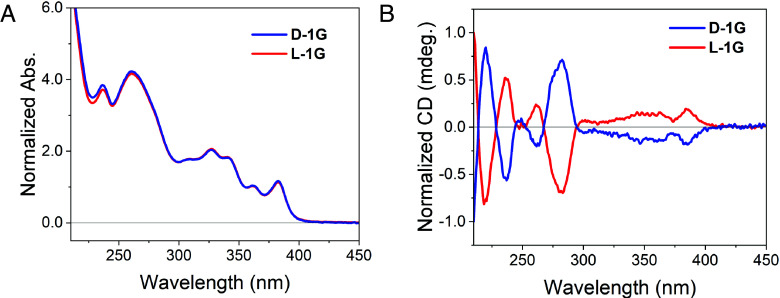
(*A*) UV-Vis absorption of **D-1G** and **L-1G** in buffer (100 mM sodium chloride and 10 mM sodium phosphate) at room temperature. (*B*) CD spectra of **D-1G** and **L-1G** in buffer (100 mM sodium chloride and 10 mM sodium phosphate) at room temperature.

The chirality of the DNA bridge was controlled using either the naturally occurring D-DNA nucleotides or the enantiomeric L-DNA nucleotides. The representative ultraviolet-visible (UV-Vis) spectra of **1G** synthesized with either D- or L-DNA are nearly identical (Fig. 2*A*). The absorption spectra show characteristic peaks for DNA (260 to 280 nm), Sd (320 nm), and NDI (355 nm, 380 nm). The circular dichroism (CD) spectra of **D-1G** and **L-1G** are equal in magnitude and opposite in sign for hairpins of equal length, indicating that **D-1G** and **L-1G** adopt configurations of right- and left-handed B-DNA helices, respectively (Fig. 2*B*).

### Charge Transfer Dynamics.

Previous work on the **D-1G**, **D-2G**, and **D-3G** hairpins provides insight into the optimal sequences to promote long-lived SCRPs with high enough quantum yields to probe using both optical and EPR techniques (26, 34). Photoexciting NDI results in rapid, exergonic hole injection into the purine sequence. When tracts of two or three adenines are used, charge recombination processes are mitigated by the formation of an A-tract polaron (34, 35). Subsequent exergonic hole transfer occurs to the guanine followed by hole trapping on Sd ensuring that the NDI^•−^-Sd^•+^ SCRP lasts several microseconds so that it can be probed with pulse-EPR techniques (26).

**Fig. 3.**
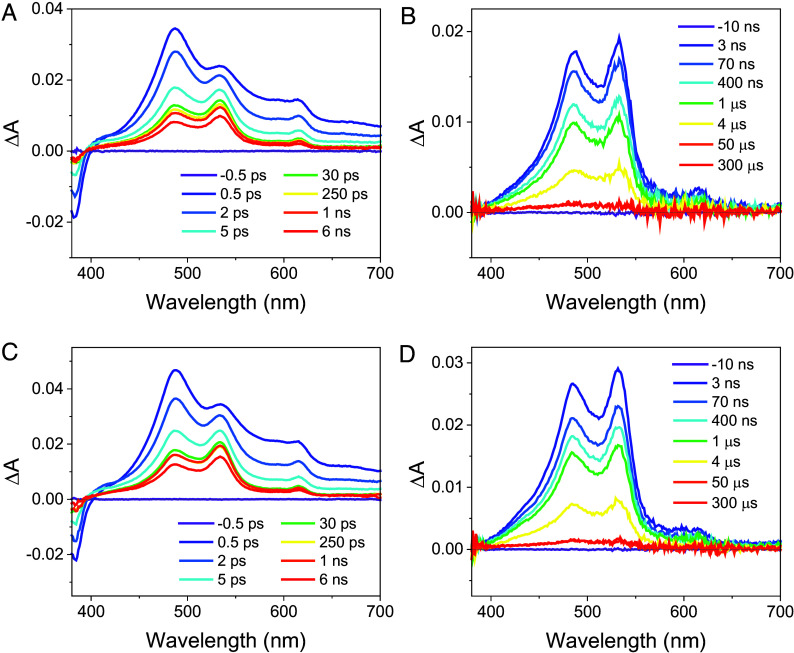
fsTA and nsTA spectra of **D-1G** (*A* and *B*), respectively, and **L-1G** (*C* and *D*), respectively, following 355 nm excitation in buffer (100 mM sodium chloride and 10 mM sodium phosphate) at room temperature at the indicated pump–probe delay times.

Femtosecond and nanosecond transient absorption (fsTA and nsTA) measurements were performed to confirm that the D-DNA and L-DNA hairpins of the same length have identical charge transfer dynamics. Representative fsTA and nsTA spectra for **D-1G** highlight important spectral features (Fig. 3). After photoexcitation, a feature at 595 nm, ascribed to ^1*^NDI (33), decays within ~300 fs, signifying rapid hole injection into the A-tract. Spin–orbit intersystem crossing (SO-ISC) to form ^3*^NDI is known to occur in several picoseconds depending on the solvent (36); however, no features characteristic of ^3*^NDI were observed at 450 nm (33, 34), meaning that hole injection is much faster than SO-ISC of ^1*^NDI. Bands at 488, 538, and 610 nm indicate the presence of NDI^•−^, accompanied at longer delay times by a feature at 535 nm that is characteristic of Sd^•+^ (33, 34). Full details of the kinetic analysis are reported in the *SI Appendix*. A comparison of the similar fsTA and nsTA spectra and kinetic behavior for the D- DNA and L-DNA hairpins indicates that the charge transfer dynamics are nearly the same for the two chiralities (*SI Appendix*, Figs. S2–S7 and Tables S2 and S3).

### TREPR Spectroscopy.

**Fig. 4.**
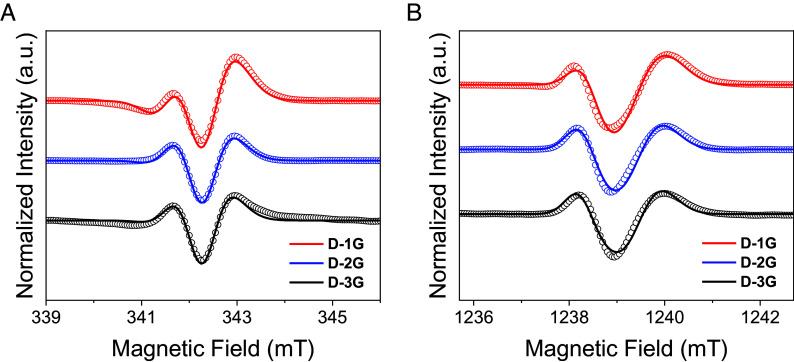
(*A*) X-band and (*B*) Q-band TREPR spectra of **D-1G**, **D-2G**, and **D-3G** in 50% glycerol/50% buffer (100 mM NaCl and 10 mM Na_3_PO_4_) 85 K, 100 ns after a 355 nm, 7 ns laser pulse. The smooth curves overlaying the experimental spectra are simulations using the parameters given in *SI Appendix*, Table S4. The field was frequency-corrected.

The TREPR spectra of **D-1G**, **L-1G**, **D-2G**, **L-2G**, **D-3G,** and **L-3G** at 85 K were obtained at X- and Q-band to evaluate the influence of CISS on SCRP formation in varying lengths of a chiral DNA bridge. Since direct detection was used, positive signals correspond to enhanced absorption (*a*) and negative signals correspond to emission (*e*). The X-band spectra of **D-1G**, **D-2G**, and **D-3G** at 100 ns after photoexcitation are shown in Fig. 4*A* and exhibit an *aea* polarization pattern for all lengths of the DNA bridge. Similarly, the Q-band spectra show an *aea* polarization pattern, although the peaks are more widely spaced (Fig. 4*B*). The TREPR spectra of **L-1G**, **L-2G**, and **L-3G** are nearly the same as those of **D-1G**, **D-2G**, and **D-3G** for the same length of DNA bridge (*SI Appendix*, Fig. S8). Thus, the sign of the chirality does not influence the spectra of these SCRPs that are randomly oriented relative to the external applied magnetic field, which is consistent with theoretical predictions (17, 18) and with our previous works on D-Bχ-A triads (20, 21).

The simulations overlaying the experimental data in Fig. 4 were obtained by fixing the spin Hamiltonian parameters and changing only the initial charge-separated state. The spin Hamiltonian for this SCRP has been described previously (21). The *g*-tensors of NDI^•+^ and Sd^•−^ are known from previous EPR measurements (26), and their relative orientation can be inferred from the spatial arrangement of the DNA helix. The spin–spin exchange (*J*) and dipolar (*D*) interactions are obtained from published out-of-phase electron spin echo envelope modulation measurements (26). The dipolar interaction dominates with a small exchange contribution only for **D-1G** and **L-1G** due to the short distance separating the two radicals. The measured *D* values agree with the values obtained from the point dipole approximation for the computed molecular structures (26). To account for the anisotropic line broadening due to the hyperfine coupling, we computed the DFT-optimized structures of NDI^•+^ and Sd^•−^, calculated their hyperfine interactions, and approximated the hyperfine-broadened spectrum using *g*-strain.

Hence, the only free parameters to fit the experimental spectra are the initial populations of the spin sublevels after hole transfer through the chiral bridge. In the typical SCRP model, we expect the initial state to be purely singlet. The CISS effect can be incorporated into the model by assuming a generic state of the form

[1] $$\left|\psi_{\chi }\right.>=\text{cos}\frac{\chi }{2}\left|S\right.>+e^{i\varphi }\text{sin}\frac{\chi }{2}\left|T_{0}(n)\right.>,$$

where $\left|S\right.>$ and $\left|T_{0}(n)\right.>$ are the singlet and triplet (*m_s_* = 0) components of the spin pair state (17, 20, 21). Note that $\left|T_{0}(n)\right.>$ is defined in terms of the orientation **n** of the molecular axis in the laboratory frame with the external field along *z*, and this must be accounted for in the simulations in a randomly oriented solution. In Eq. **1**, the CISS efficiency is quantified by the angle χ, ranging from 0 for a 0% CISS contribution to ± π/2 for a 100% CISS contribution with opposite signs for each enantiomer. The CISS contribution is then given by $p=2\;{\text{sin}}^{2}\frac{\chi }{2}$, with 0 ≤ *p* ≤ 1). Note that hole injection to the adenine has been established to far outcompete SO-ISC of ^1*^NDI kinetically at 85 K; (34) hence, all spectral features that cannot be explained by a singlet precursor radical pair model are attributed to the CISS effect.

It is important to note that TREPR spectroscopy on molecules in a randomly oriented powder or aligned with a liquid crystal only gives access to *p* and not the coherence between $\left|S\right.>$ and $\left|T_{0}(n)\right.>$ and in particular to the phase φ. Hence, it cannot discriminate an initial state of the form $\left|\psi_{\chi }\right.>$ with real off-diagonal matrix elements (17) from one with purely imaginary off-diagonal matrix elements (37), or from an incoherent mixture of $\left|S\right.>$ and $\left|T_{0}(n)\right.>$.

**Fig. 5.**
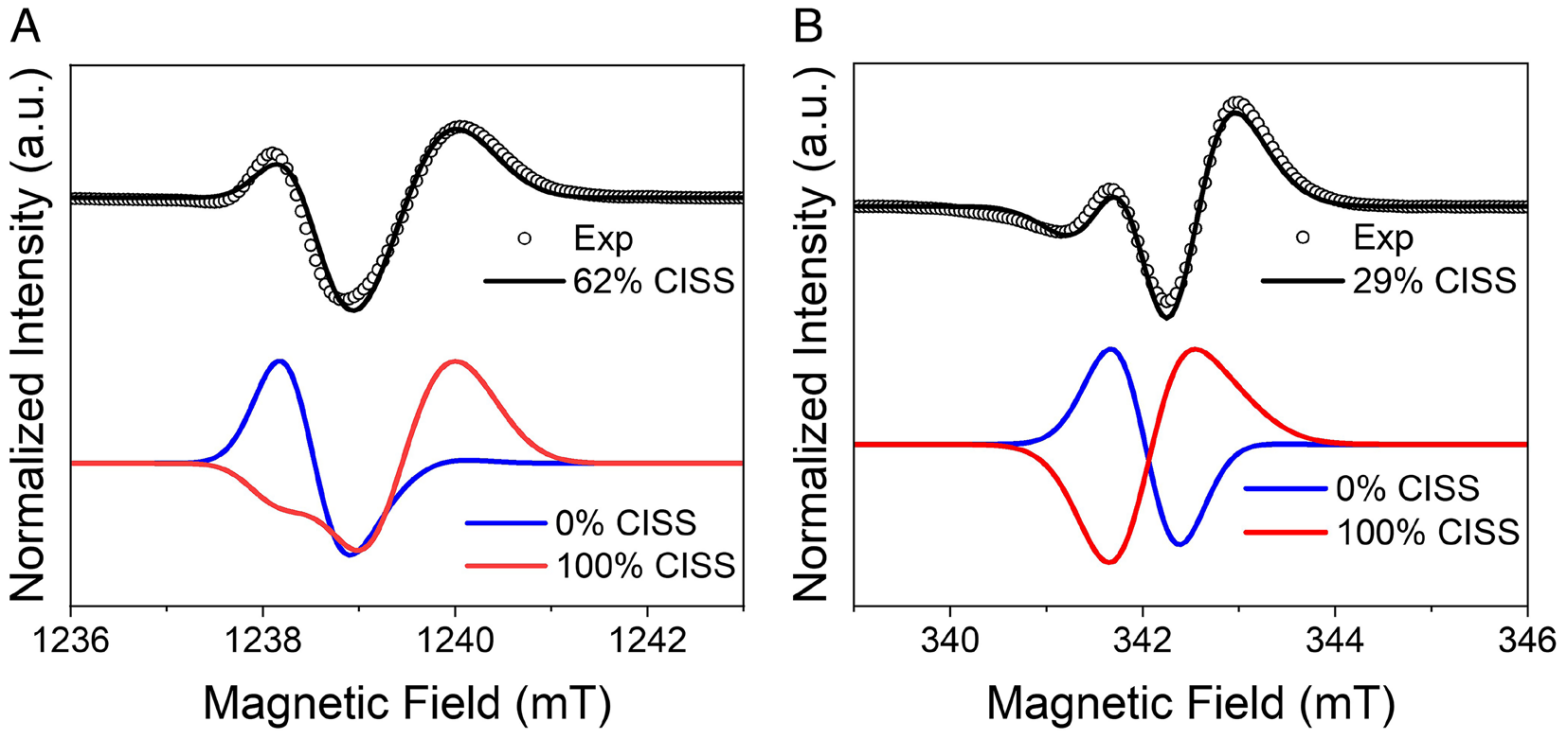
(*A*) Q-band and (*B*) X-band TREPR spectra of **D-1G** in 50% glycerol/50% buffer (100 mM NaCl and 10 mM Na_3_PO_4_) 85 K, 100 ns after a 355 nm, 7 ns laser pulse (black circles). The smooth curves overlaying the experimental spectra are simulations using the parameters given in *SI Appendix*, Table S4 with CISS contributions of 0% (blue curve), 100% (red curve), or the optimal fit (black curve).

To illustrate the influence of CISS on the TREPR spectra, we consider the **D-1G** hairpin as an example. Fig. 5 *A*, *Top* shows the TREPR spectrum of **D-1G** collected at Q-band (black circles) with the simulation (black line) computed using the limits illustrated in Fig. 5 *A*, *Bottom*, where the simulated spectrum for a pure singlet (blue curve) and pure CISS-derived triplet (red curve) are shown. The simulation for the pure singlet state shows a low-field absorptive feature at ~1,238 mT and no absorptive feature at ~1,240 mT. However, the experimental spectrum has a pronounced absorptive feature at ~1,240 mT, which correlates with the dominant absorptive feature in the pure CISS case. Hence, only intermediate values of the angle χ corresponding to a combination of the two traces can reproduce the experimental data, leading to the optimal fit of 62% CISS represented by the black curve superimposed on the black circles in Fig. 5*A*. This shows unambiguously that the precursor state cannot be a pure singlet and hence demonstrates the occurrence of CISS, which imparts partial triplet character to the SCRP. For the Q-band spectra, the relative intensity of the two maxima is a direct measure of the CISS contribution to NDI^•−^-Sd^•+^ (*SI Appendix*, Fig. S10).

In the X-band TREPR spectrum of **D-1G** (Fig. 5*B*), the peaks are closer, which leads to a partial cancellation of singlet and CISS contributions. Nonetheless, the influence of CISS is still clear from comparing the relative intensity of the two maxima, leading to a CISS contribution of 29%. The CISS contribution measured at X-band is sensitive to other parameters such as peak broadening and *g*-strain. Nevertheless, the measured CISS contribution remains within the ±5% error bars (*SI Appendix*, Fig. S12).

Table 1 shows the average percentage of CISS used in simulating the TREPR at X- and Q-band for **D-1G**, **L-1G**, **D-2G**, **L-2G**, **D-3G,** and **L-3G**. While the trend seems to be slightly increasing at X-band and slightly decreasing at Q-band, the values are still within experimental error. Hence, we do not find a significant dependence of the CISS contribution on the number of C-G base pairs in the DNA bridge. This may be a result of charge (spin) delocalization in the G-tract in the intermediate step that produces NDI^•−^- G^•+^ as was reported earlier (26). This is consistent with a recent model for CISS in photoinduced electron transfer for a similar two-step electron transfer process, in which charge delocalizes on the bridge before transferring to the charge trap (23).

**Table 1.** Average CISS percentages (±5%) used to fit TREPR spectra in Fig. 5 for the indicated DNA hairpins

DNA Hairpin	% CISS at X-Band	% CISS at Q-Band
**D-1G**	29	62
**D-2G**	33	52
**D-3G**	35	47
**L-1G**	28	61
**L-2G**	32	53
**L-3G**	32	50

Interestingly, we find that the CISS contribution increases significantly from X- to Q-band, showing an apparent magnetic field dependence. Such a dependence of *p* (or, equivalently, of the angle χ in Eq. **1**) on *B* goes beyond the simple spectral change due to the loss of coherences between eigenstates and implies a field-dependent initial state observed by TREPR spectroscopy. A possible way to obtain a magnetic field dependence of the triplet component without invoking CISS contribution would be to consider the possibility of coherent singlet-triplet mixing occurring in the intermediate NDI^•−^-G^•+^ radical pair before the formation of the final NDI^•−^-Sd^•+^ state (38). This depends critically on the lifetime of NDI^•−^-G^•+^ as well as its magnetic parameters. While it is clear that *J* is small and *D* is modest for NDI^•−^-G^•+^ because of the long distances between the two radicals, its lifetime may be too short for significant coherent spin evolution to occur (34). To address this issue, we simulated the possible contribution of $|S>-|T_{0}>$ mixing within NDI^•−^-G^•+^ to that observed in NDI^•−^-Sd^•+^ using the model proposed by Hore (38) (*SI Appendix*). Given the magnetic parameters of the DNA hairpins (*SI Appendix*, Table S4 and ref. 39) and the short NDI^•−^-G^•+^ lifetime (34), the triplet contribution of NDI^•−^-G^•+^ via the radical pair intersystem crossing mechanism to the observed TREPR spectra of NDI^•−^-Sd^•+^ is insignificant (*SI Appendix*, Fig. S13).

**Fig. 6.**
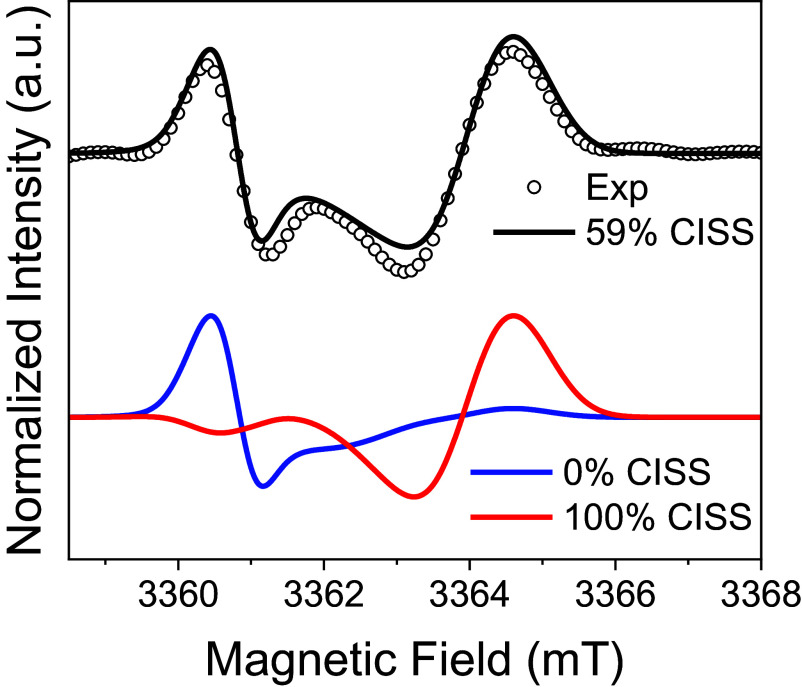
W-band EDFS of **D-1G** in 50% glycerol/50% buffer (100 mM NaCl and 10 mM Na_3_PO_4_) 85 K, 150 ns after a 355 nm, 7 ns laser pulse (black circles). The smooth curves overlaying the experimental spectra are simulations using the parameters given in *SI Appendix*, Table S4 with CISS contributions of 0% (blue curve), 100% (red curve), or the optimal fit (black curve).

An increase of local spin polarization with *B* may occur as a result of relaxation of the bridge states involved in formation of the SCRP, as considered in ref. 23. This bridge relaxation process is complete well before the time that the EPR measurements are made and results in an increase of triplet character of the SCRP (*SI Appendix*) with *B*. Indeed, in this model, the bridge state relaxation involves the SCRP eigenstates, which show a strong dependence on the ratio between $\Delta g\mu_{B}B$ and *D*. Thus, the increase in the observed triplet character of the SCRP with *B* should occur up to a certain saturation field, because once $\Delta g\mu_{B}B\gg D$, the SCRP eigenstates are not significantly modified by further increasing *B*. Hence, we expect the longer hairpins, which are characterized by smaller dipolar couplings, to reach saturation for smaller values of *B*, consistent with previous reports for systems with small *D* (21). To corroborate these ideas, we performed W-band, echo-detected TREPR measurements on **D-1G** (Fig. 6). A CISS contribution of 59 ± 5% is needed to reproduce the experimental spectrum, comparable to that observed at Q-band. This suggests that we have already reached the saturation condition $\Delta g\mu_{B}B\gg D$ at Q-band, where *B* ≅ 1.2 T. However, the observed magnetic field dependence of the CISS contribution is not reproduced by the mechanism outlined in the *SI Appendix* and in ref. 23 alone, because the predicted increase in local spin polarization is not necessarily accompanied by an increase of CISS contribution when coherences between eigenstates are lost (40). Hence, additional theory will be needed to properly model this effect.

## Conclusions

We observe signatures of CISS in the TREPR spectra of **D-1G**, **L-1G**, **D-2G**, **L-2G**, **D-3G,** and **L-3G**, showing that DNA helices can impart triplet character to the SCRP formed during hole transfer in chromophore-linked hairpins. We observed no notable dependence of CISS on DNA length, most probably due to hole delocalization over the G-tract. Furthermore, we noted an apparent increase in CISS contribution to the TREPR spectra with magnetic field, which could reflect the magnetic field dependence of SCRP eigenstates and needs to be considered in future models of CISS in charge transfer. Leveraging the chirality of DNA to control quantum states is an exciting prospect for future quantum information applications.

## Materials and Methods

### Synthesis, Purification, and Characterization.

The DNA hairpins were synthesized by standard solid-phase oligonucleotide synthesis methods using a K&A H-2 synthesizer. Commercially available D-DNA and L-DNA phosphoramidites were used to modify the chirality of the hairpins. The preparation of the chromophores *N*,*N*′-[bis(3-hydroxypropyl)]-naphthalene-1,4:5,8-bis(dicarboximide) (NDI) (41) and bis(2-hydroxyethyl)stilbene-4,4′-diether (Sd) (42) have been reported previously. Both the NDI and Sd diols were converted to monosubstituted dimethoxytrityl derivatives, followed by conversion to the cyanoethyl-*N*,*N*-diisopropyl phosphoramidite derivatives, allowing them to be incorporated into the hairpins. DNA hairpins were purified using reverse-phase high-performance liquid chromatography and characterized by matrix-assisted laser desorption ionization–time of flight mass spectrometry, UV-vis spectroscopy, and CD spectroscopy. Additional details are available in *SI Appendix*.

### TA Spectroscopy.

fsTA and nsTA spectra for each hairpin were collected at room temperature using a commercial Ti:sapphire laser system (Tsunami oscillator/Spitfire amplifier, Spectra-Physics) which has been described previously (43). Samples (100 to 150 μM) were suspended in a buffer containing 10 mM sodium phosphate and 100 mM sodium chloride to achieve an optical density (OD) of 0.2 to 0.5 in a 2 mm cuvette. The solutions were degassed by bubbling nitrogen through septa-capped cuvettes for 20 min and stirred during the measurements. A commercial collinear optical parametric amplifier (TOPAS-Prime, Light-Conversion, LLC) was used to generate 355 nm pump pulses. The pump pulses were attenuated to 1 μJ/pulse and depolarized to reduce polarization-dependent dynamics. The TA spectra were detected using a customized Helios/EOS spectrometer and Helios software (Ultrafast Systems, LLC). The Surface Xplorer software (Ultrafast Systems, LLC) was used to remove scattered light and chirp correct the raw data. The kinetic analysis was performed using a home-written MATLAB program following a multiexponential decay model.

### TREPR Spectroscopy.

EPR measurements were performed on DNA hairpins (200 to 250 μM) in 50% aqueous buffer (100 mM sodium chloride and 10 mM sodium phosphate buffer) and 50% glycerol with an OD of 0.5 to 1.0 at 355 nm in a 2 mm cuvette. Solutions were loaded into quartz tubes (Vitrocom) of 2.40 mm o.d., 2.00 mm i.d. for X-band, 1.50 mm o.d., 1.00 mm i.d. for Q-band, and 0.86 o.d., 0.6 i.d. for W-band measurements. The samples were flash-frozen in liquid nitrogen and loaded into the precooled resonator at 85 K. The X- and W-band measurements were obtained on a Bruker Elexsys E680 X/W EPR spectrometer with a split ring resonator (Bruker ER4118X-MS3) at X-band and a cylindrical resonator (Bruker EN-680-1021H) at W-band. The Q-band measurements were performed on a home-built instrument described previously (21). The temperature was maintained at 85 K using an Oxford Instruments CF935 continuous-flow optical cryostat with liquid nitrogen. Samples were photoexcited at 355 nm (1.5 mJ/pulse, 7 ns, 10 Hz) from the frequency-tripled output of a Nd:YAG laser.

TREPR spectra were collected at X- and Q-band and processed according to previously published procedures (20). Due to spectrometer limitations, at W-band, the TREPR was collected with a two-pulse Hahn echo sequence at a fixed time of *t* = 150 ns following photoexcitation. using a hυ-t-π/2-τ-π-echo pulse sequence. Pulse lengths of π/2 = 20 ns, π = 40 ns and τ=300 ns were used to collect the full transient trace of the echo at each field both with and subsequently without photoexcitation. The dark spectrum was subtracted from the light spectrum and plotted. All processing and fitting of the spectra were performed in MATLAB using home-written scripts and the simulation package EasySpin v6.0-dev.5411 (44, 45).

## Supplementary Material

Appendix 01 (PDF)

## Data Availability

The data that support the findings of this study are found in the *SI Appendix*.
