# Supplementary material for: Detecting chirality-induced spin selectivity in chromophore-linked DNA hairpins using photogenerated radical pairs
Source: Proc Natl Acad Sci U S A. 2025 Aug 5;122(32):e2515120122. doi: 10.1073/pnas.2515120122 (PMC12358874; doi:10.1073/pnas.2515120122)
Supplement: Supplementary file 1 — Appendix 01 (PDF) [file pnas.2515120122.sapp.pdf]

Supporting Information Appendix for

Detecting Chirality-Induced Spin Selectivity in Chromophore-Linked DNA Hairpins using Photogenerated Radical Pairs

Elisabeth I. Latawiec,[1] Alessandro Chiesa,[2] Yunfan Qiu,[1] Nikolai A. Tcyrulnikov,[1] Ryan M. Young,[1] Stefano Carretta,[2*] Matthew D. Krzyaniak,[1*] and Michael R. Wasielewski[1*]

[1]Department of Chemistry, Institute for Quantum Information Research and Engineering, and Center for Molecular Quantum Transduction, Northwestern University, Evanston, IL 60208-3113 United States

[2]Università di Parma, Dipartimento di Scienze Matematiche, Fisiche e Informatiche, Parma, I-43124, Italy; INSTM, UdR Parma, I-43124 Parma, Italy; INFN Sezione Milano-Bicocca, Gruppo Collegato di Parma, I-43124 Parma, Italy;

*to whom correspondence should be addressed. Email: m-wasielewski@northwestern.edu; mdkrzyaniak@northwestern.edu; stefano.carretta@unipr.it

Contents

Synthesis **2**
UV-Vis, Circular Dichroism, and MALDI Characterization **4**
Transient Absorption Spectroscopy **5**
Electron Paramagnetic Resonance Spectroscopy **13**
Additional X-Band Data Analysis **15**
Relaxation Theory and Magnetic Field Dependence **17**
DFT Calculations of EPR Parameters **21**
References **23**

Synthesis

Materials. Chemicals were purchased as reagent grade and used as received. naphthalene-1,4,5,8-tetracarboxydianhydride (NDA, TCI, ≥ 97%), 3-amino-1-propanol (3-AMP, Sigma-Aldrich, ≥ 99%), zinc acetate dihydrate (Sigma-Aldrich, ≥ 99%), anhydrous pyridine, 4,4'-dimethoxytrityl chloride (DMT chloride, ChemeGenes), *N,N*-diisopropylethylamine (DIPEA, Sigma-Aldrich, 99.5%), and 2-cyanoethyl *N,N*-diisopropylchlorophosphoramidite (Acros, 97%). DNA synthesis reagents were purchased from Glen Research: Oxidizing Solution (0.02 M iodine in tetrahydrofuran/water/pyridine), Cap Mix A (tetrahydrofuran/acetic anhydride), Cap Mix B (10% 1-methylimidazole in tetrahydrofuran/pyridine), Activator (Sublimed 1H-Tetrazole in Anhydrous Acetonitrile), Deblocking Mix (3% trichloroacetic acid in dichloromethane), dA-CE phosphoramidite, dG-CE phosphoramidite, dT-CE phosphoramidite, dC-CE phosphoramidite, beta-L-iPr-Pac-dG-CE phosphoramidite, beta-L-Pac-dA-CE phosphoramidite, beta-L-Ac-dC-CE phosphoramidite, beta-L-dT-CE phosphoramidite, dC-CPG 500, and Glen UnySupport™ 500.

Chromophore Synthesis. Both the bis(2-hydroxyethyl)stilbene-4,4′-diether (Sd) and *N,N′*-[bis-(3-hydroxypropyl)]-naphthalene-1,4:5,8-bis(dicarboximide) (NDI) chromophores were synthesized following previously reported procedures.[1] For each chromophore, the diol precursor was subjected to the addition of dimethoxytrityl followed by conversion to the 2-cyanoethyl-*N,N*-diisopropylphosphoramidite derivatives as previously described.

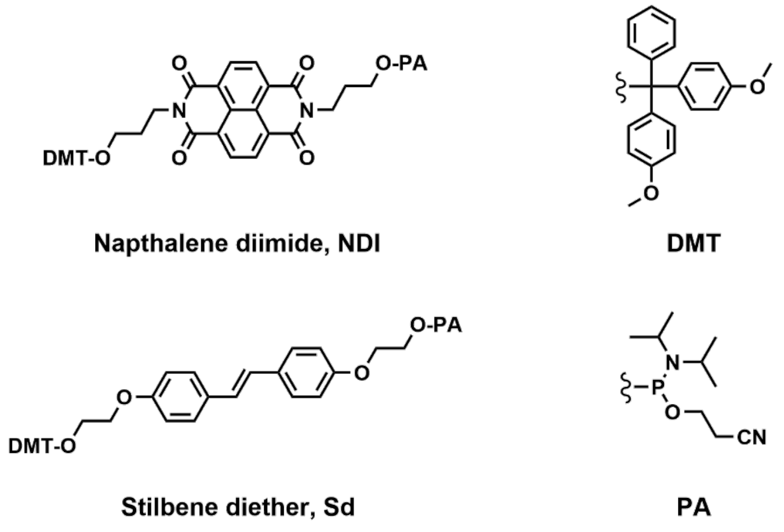

Fig. S1. Structures of NDI and Sd derivatives used for DNA synthesis.

DNA Synthesis and Purification. The DNA hairpins were synthesized using a K&A H-2 synthesizer. A standard 1 μmol DNA synthesis protocol was used with 50 mg of either dC-CPG for the D-hairpins or universal support for the L-hairpins. The functionalized NDI or Sd chromophore derivatives were loaded onto the DNA synthesizer as 0.15 M solutions in anhydrous acetonitrile.

The oligonucleotides were synthesized with the terminal DMT removed and were stored on CPG in a freezer until purification. For the D-hairpins, the CPG bound oligos were treated with ammonium hydroxide solution for 36 hours at room temperature to deprotect the bases and remove the DNA from the CPG. For the L-hairpins, 50 mM potassium carbonate in methanol was used instead of ammonium hydroxide. After filtering and evaporating the deprotection solution, the DNA conjugates were purified by HPLC on a C-18 reverse phase column (250 x 10.0 mm, Phenomenex) with a 2% per minute gradient of acetonitrile in 0.03 M aqueous triethylammonium acetate. The product was collected at ~17 mins and dried under nitrogen. The product was further

purified by HPLC using a 1% gradient of acetonitrile in 0.03 M aqueous triethylammonium acetate, collecting at ~24 mins. The purity of the final product was confirmed by MALDI-TOF.

UV-Vis, Circular Dichroism, and MALDI Characterization

CD Spectroscopy. CD spectra were acquired on a commercial spectrometer (Jasco, J-1700). DNA hairpins were suspended in a buffer containing 10 mM sodium phosphate and 100 mM sodium chloride in a 2 mm cuvette. Spectra were corrected by subtracting the buffer spectrum and normalized at 355 nm.

UV-Vis Spectroscopy. Absorption spectra were acquired on the CD spectrometer listed above. DNA hairpins were suspended in a buffer containing 10 mM sodium phosphate and 100 mM sodium chloride in a 2 mm cuvette. Spectra were corrected by subtracting the buffer spectrum and normalized at 355 nm.

MALDI-TOF mass spectrometry. Samples were mixed with a 2,5-dihydroxyacetophenone (DHAP) matrix. A Bruker AutoFlex-III instrument was used in linear negative mode.

Table S1. MALDI-TOF mass spectrometry

**Hairpin**	**Expected mass (m/|z|)**	**Experimental mass (m/|z|)**
**D-1G**	3215.4	3215.6
**D-2G**	3833.8	3834.5
**D-3G**	4452.0	4452.3
**L-1G**	3215.4	3215.0
**L-2G**	3833.8	3834.1
**L-3G**	4452.0	4453.0

Transient Absorption Spectroscopy

Experimental Details. Details of the transient absorption instrumentation have been described previously.[2] Briefly, ~50% of the output of a 1 kHz amplified Ti:sapphire system at 827 nm (1 W, 100 fs, Spitfire, Spectra Physics) is used to pump a non-collinear optical parametric amplifier (TOPAS-White, Light-Conversion, LLC.) tuned to generate the ~60 fs, 355 nm pump pulses. The pump is depolarized to minimize polarization-specific dynamics. In the nsTA experiment, the probe is generated in a separately delayed broadband laser system (EOS, Ultrafast Systems, LLC). The transmitted probe is detected on a commercial spectrometer (customized Helios-EOS, Ultrafast Systems, LLC). Samples were stirred in 2 mm quartz cuvettes during acquisition to minimize the effects of local heating and sample degradation.

Femtosecond and nanosecond transient absorption spectra of **D-1G**, **L-1G**, **D-2G**, **L-2G**, **D-3G** and **L-3G** are shown in Figs. S2-S7. The prominent optical signatures for NDI anion (488 nm) and Sd cation (538 nm) were fit using a multiexponential decay model. The kinetic traces exhibit multi-exponential kinetics because of the complex nature of charge transfer and recombination in DNA that contains several degenerate sites where the hole can reside. However, by approximately quantifying the lifetimes of the charge separated states (Tables S2 and S3), we can verify that the L- and D-DNA exhibit the same charge dynamics for a given DNA sequence.

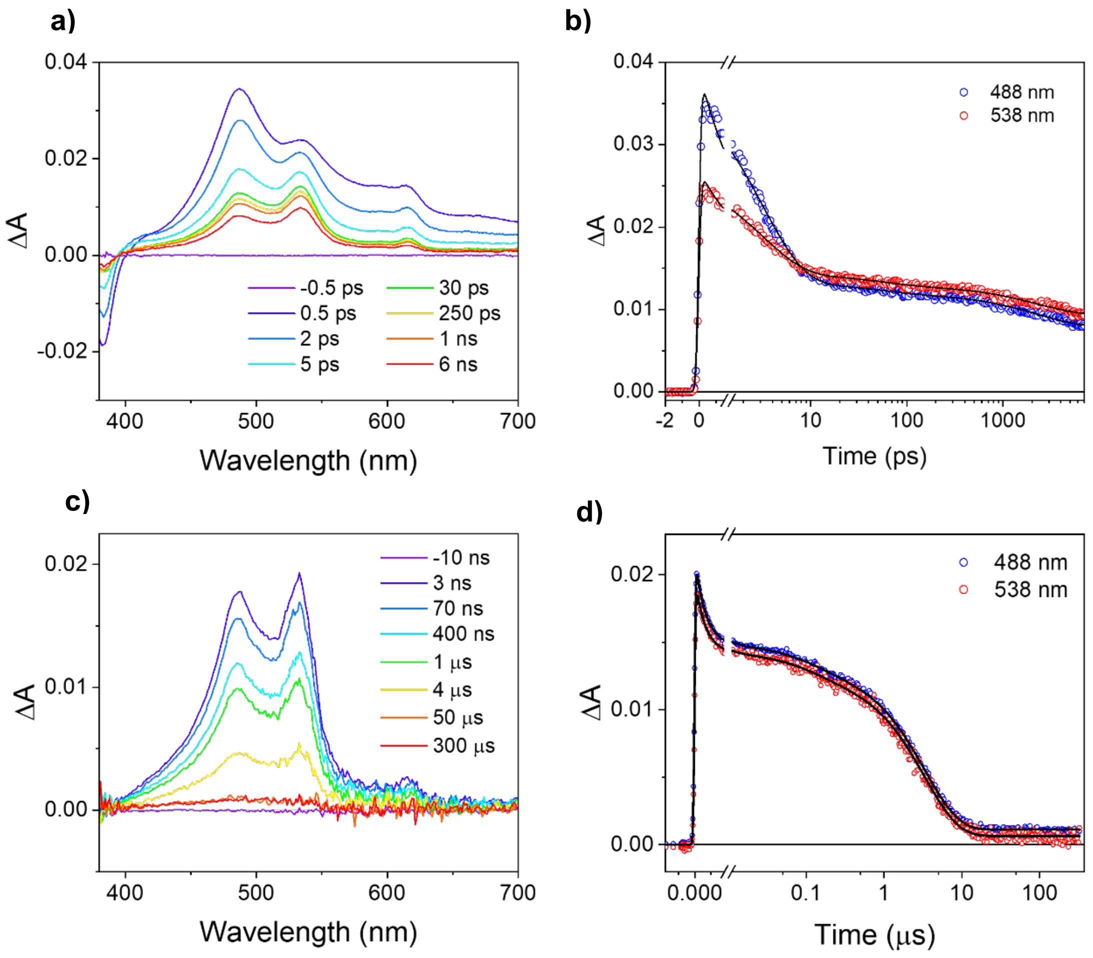

Fig. S2. (a) Femtosecond and (c) nanosecond transient absorption spectra of **D-1G** following 355 nm excitation in buffer (100 mM sodium chloride and 10 mM sodium phosphate) at room temperature. (b) Femtosecond and (d) nanosecond kinetic traces and fits.

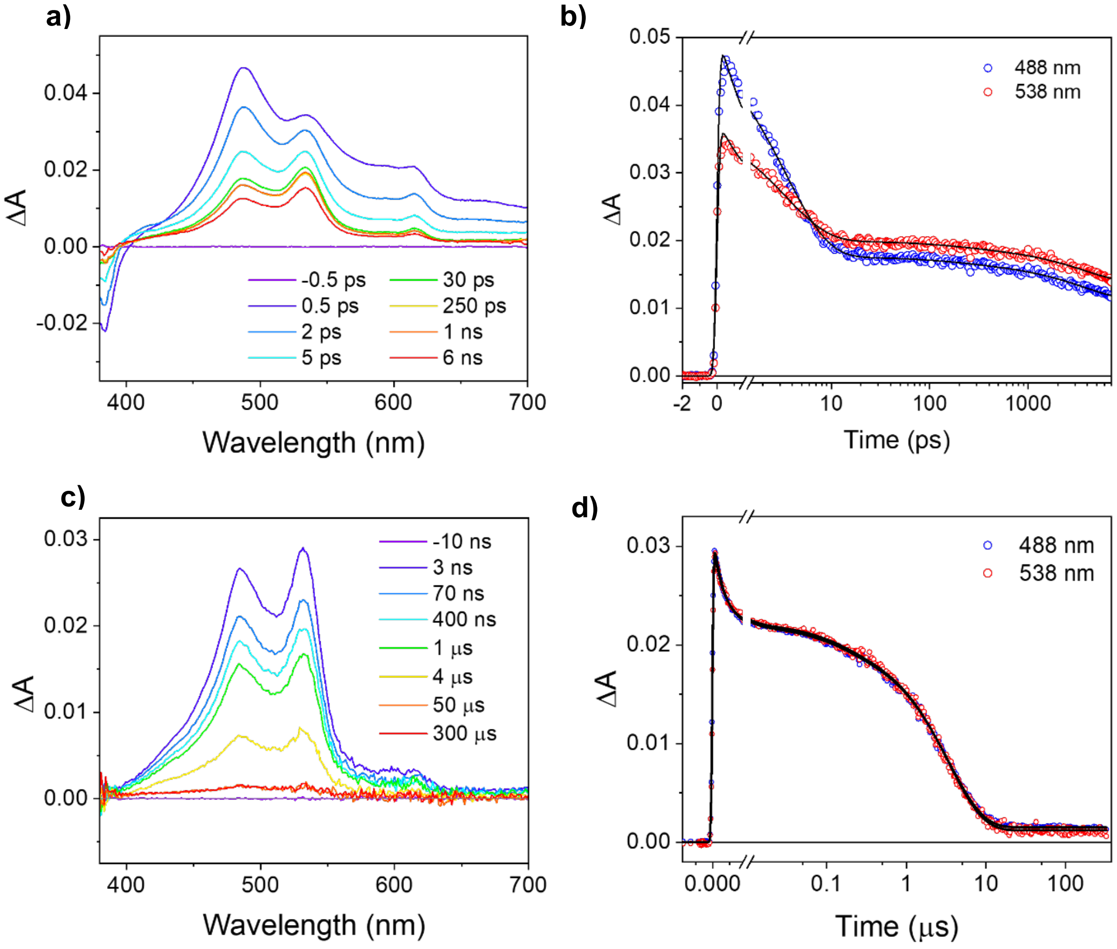

Fig. S3. (a) Femtosecond and (c) nanosecond transient absorption spectra of **L-1G** following 355 nm excitation in buffer (100 mM sodium chloride and 10 mM sodium phosphate) at room temperature. (b) Femtosecond and (d) nanosecond kinetic traces and fits.

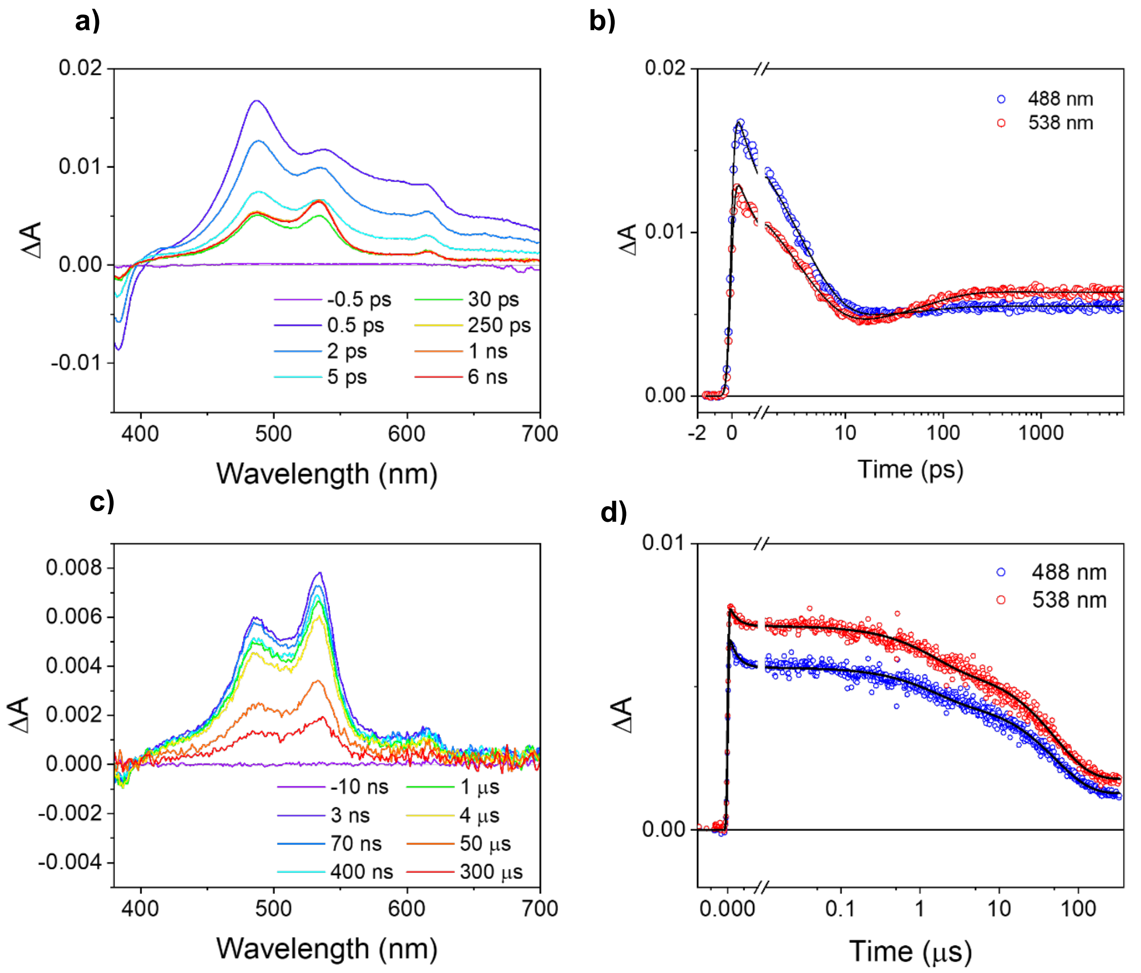

Fig. S4. (a) Femtosecond and (c) nanosecond transient absorption spectra of **D-2G** following 355 nm excitation in buffer (100 mM sodium chloride and 10 mM sodium phosphate) at room temperature. (b) Femtosecond and (d) nanosecond kinetic traces and fits.

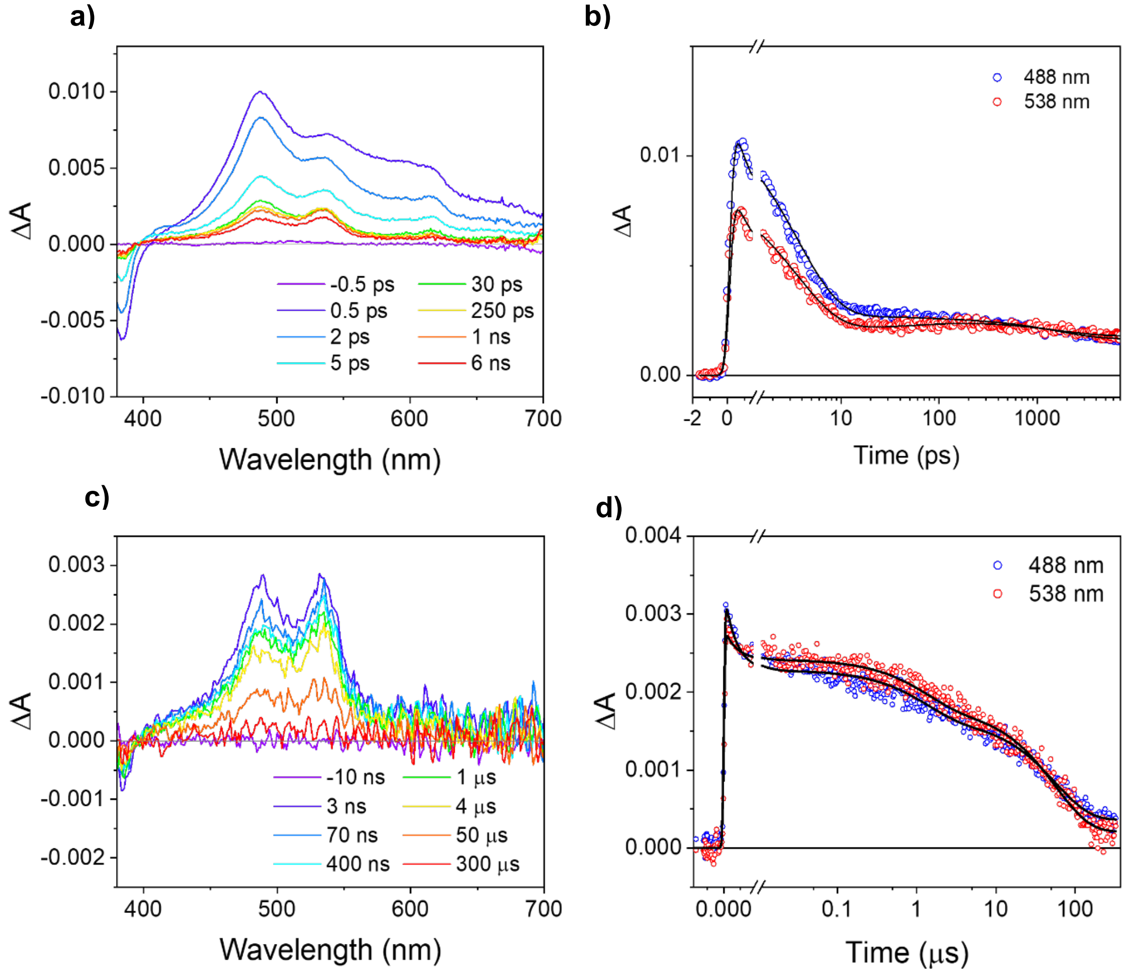

Fig. S5. (a) Femtosecond and (c) nanosecond transient absorption spectra of **L-2G** following 355 nm excitation in buffer (100 mM sodium chloride and 10 mM sodium phosphate) at room temperature. (b) Femtosecond and (d) nanosecond kinetic traces and fits.

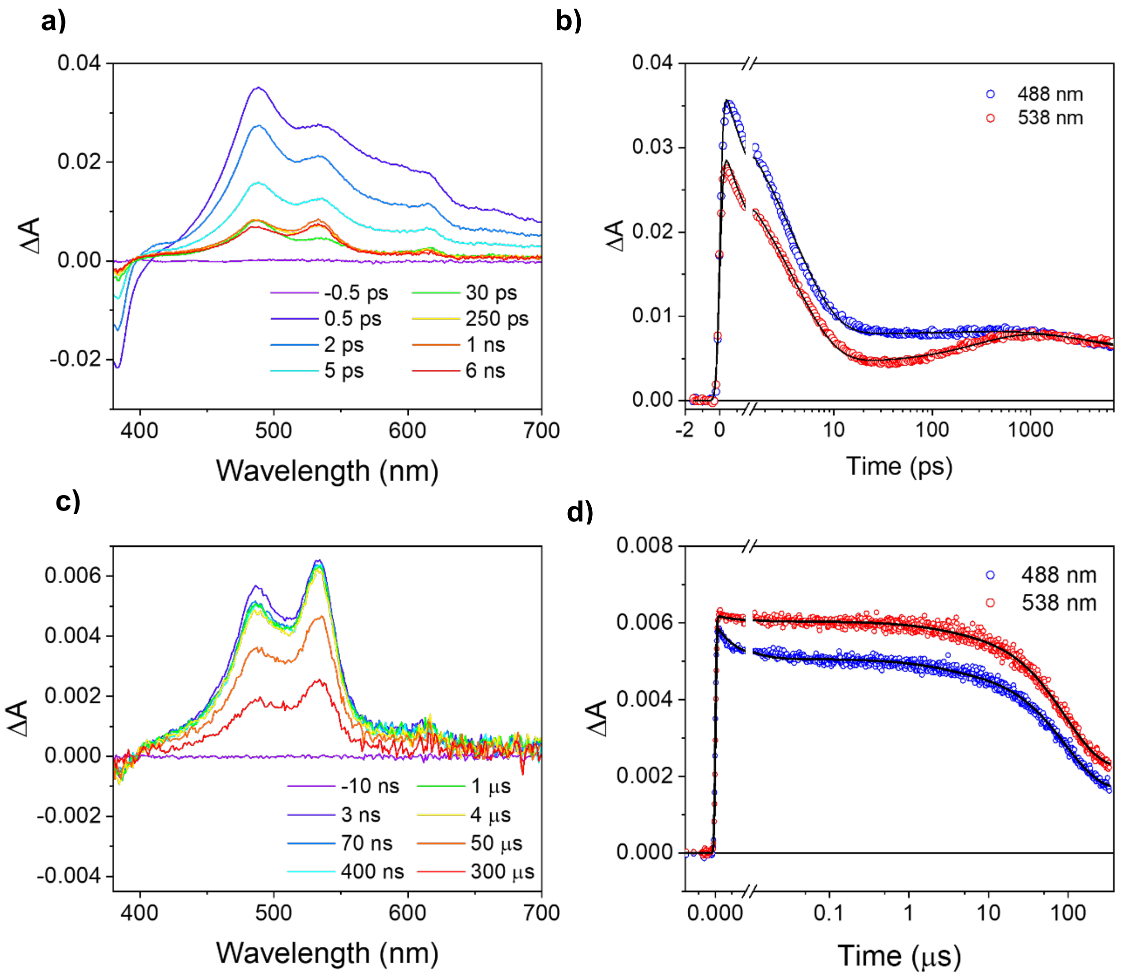

Fig. S6. (a) Femtosecond and (c) nanosecond transient absorption spectra of **D-3G** following 355 nm excitation in buffer (100 mM sodium chloride and 10 mM sodium phosphate) at room temperature. (b) Femtosecond and (d) nanosecond kinetic traces and fits.

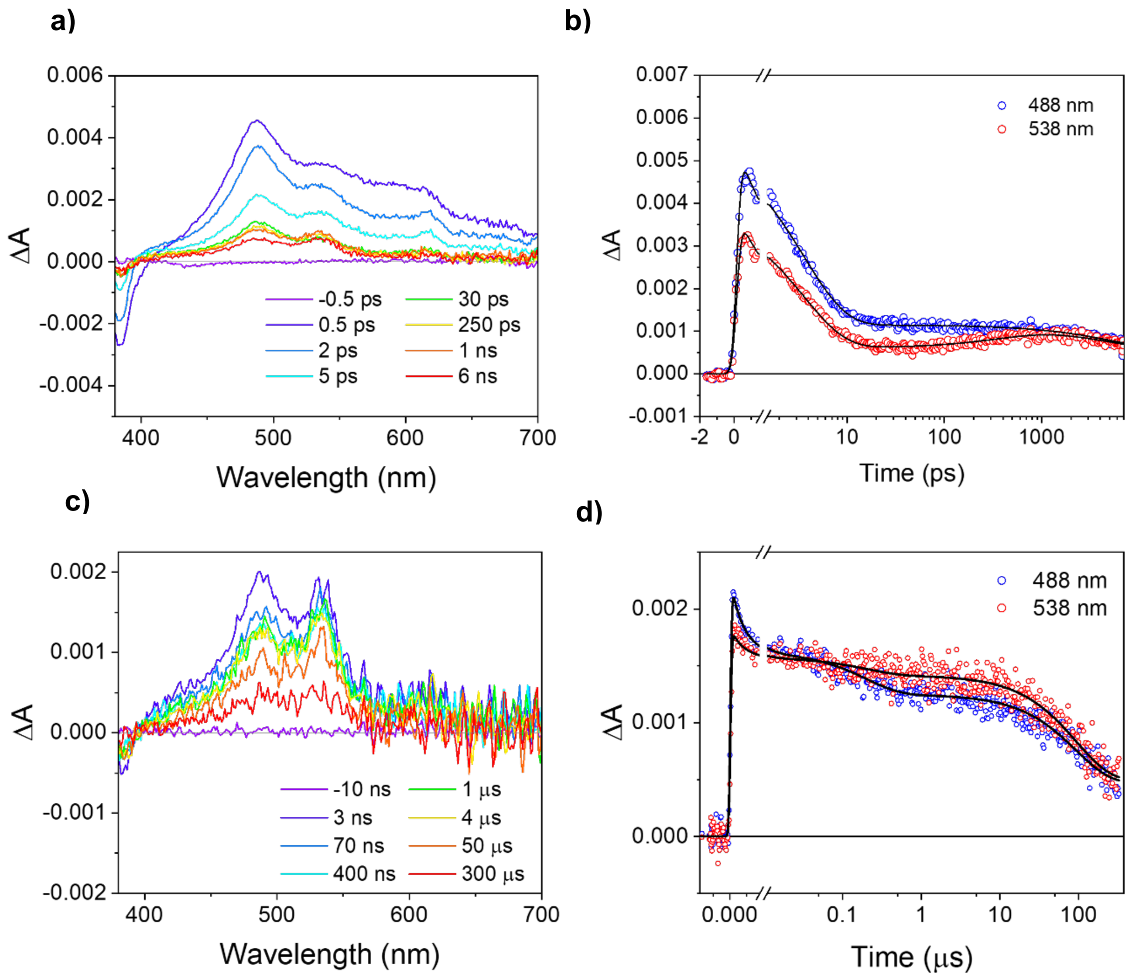

Fig. S7. (a) Femtosecond and (c) nanosecond transient absorption spectra of **L-3G** following 355 nm excitation in buffer (100 mM sodium chloride and 10 mM sodium phosphate) at room temperature. (b) Femtosecond and (d) nanosecond kinetic traces and fits.

Table S2. Time constants extracted from femtosecond transient absorption kinetic fits

Hairpin	τ_1 (ps)	τ_2 (ps)	τ_3 (ns)	τ_4 (ns)
**D-1G**	3.2 ± 0.3	55 ± 9	2.3 ± 0.2	> 7
**D-2G**	3.4 ± 0.3	67 ± 4	1.2 ± 1	> 7
**D-3G**	3.9 ± 0.3	310 ± 30	4.2 ± 1	> 7
**L-1G**	3.4 ± 0.3	200 ± 80	3.8 ± 0.7	> 7
**L-2G**	3.6 ± 0.3	70 ± 20	1.6 ± 0.2	> 7
**L-3G**	3.6 ± 0.6	420 ± 70	4.9 ± 2	> 7

Table S3. Time constants extracted from nanosecond transient absorption kinetic fits

Hairpin	τ_1 (ns)	τ_2 (μs)	τ_3 (μs)	τ_4 (μs)
**D-1G**	3.5 ± 0.6	0.117 ± 0.007	3.34 ± 0.02	> 330
**D-2G**	2.7 ± 0.6	1.40 ± 0.02	52.0 ± 0.8	> 330
**D-3G**	6.6 ± 0.6	2.4 ± 0.3	93 ± 1	> 330
**L-1G**	3.9 ± 0.6	0.146 ± 0.007	3.41 ± 0.02	> 330
**L-2G**	4.0 ± 0.6	1.29 ± 0.07	57 ± 2	> 330
**L-3G**	4.3 ± 0.6	0.200 ± 0.02	94 ± 4	> 330

Electron Paramagnetic Resonance Spectroscopy

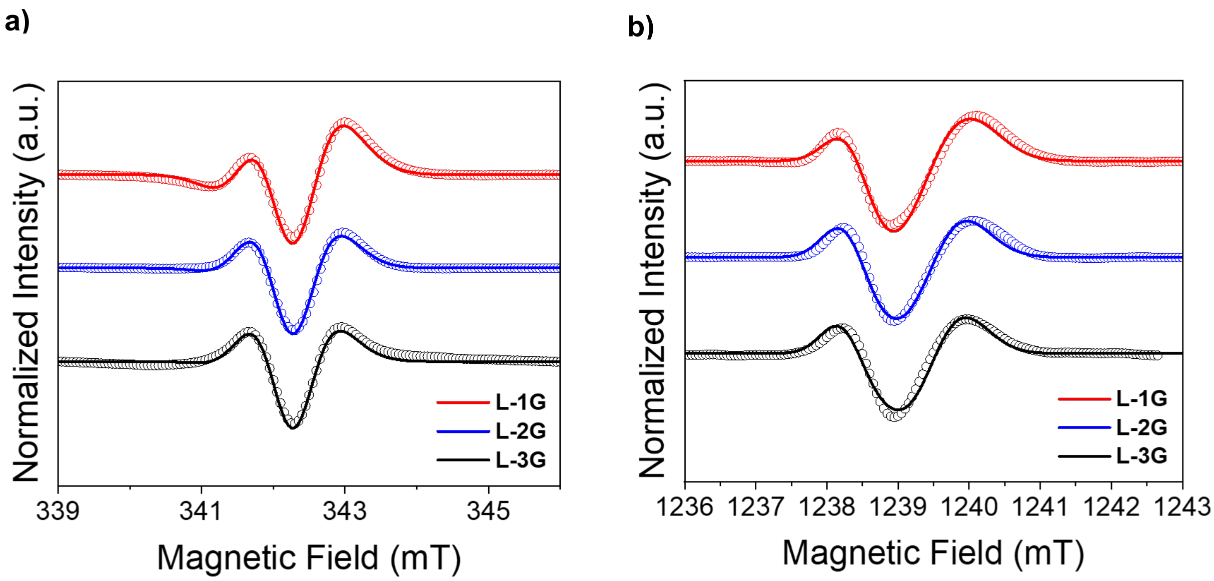

Fig. S8. (a) X-band and (b) Q-band TREPR spectra of **L-1G**, **L-2G**, and **L-3G** in 50% glycerol/50% buffer (100 mM NaCl and 10 mM Na_3PO_4) 85 K, 100 ns after a 355 nm, 7 ns laser pulse. The smooth curves overlaying the experimental spectra are simulations using the parameters given in Table S4. The field was frequency-corrected.

Table S4. TREPR simulation parameters

g-tensors	NDI$^{\bullet -}$: [2.0044 2.00467 2.0022]<br>Sd$^{\bullet +}$: [2.0033 2.0043 2.00235]
Distance (nm)	**1G**: 1.7<br>**2G**: 2.04<br>**3G**: 2.38
J (MHz)	**1G**: 0.2<br>**2G**: 0.005<br>**3G**: 0
lwpp (mT)	X: 0.5<br>Q: 0.4<br>W: 0.4
gStrain	X: [0.00313 0.00328 0.00424;<br>0.002 0.002 0.007];<br>Q: [0.00075 0.00075 0.00128;<br>0.00136 0.00045 0.00145];<br>W: [0.0001 0.0003 0.0006;<br>0.0015 0.001 0.0005];
gFrame	1G: [0 0 0; (34*5) 0 0]*pi/180;<br>2G: [0 0 0; (34*6) 0 0]*pi/180;<br>3G: [0 0 0; (34*7) 0 0]*pi/180;

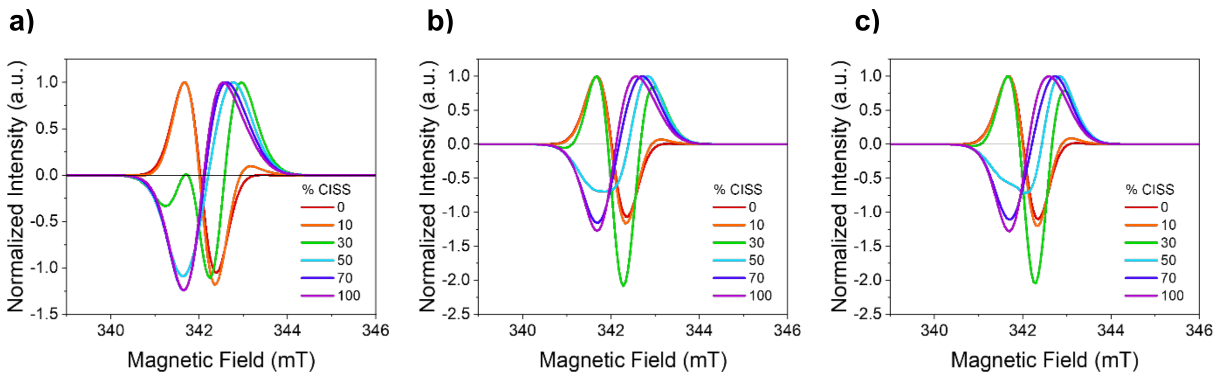

Fig. S9. X-band TREPR simulations with varying contributions of CISS for (a) **1G** (b) **2G** and (c) **3G.** Simulation parameters are given in Table S4.

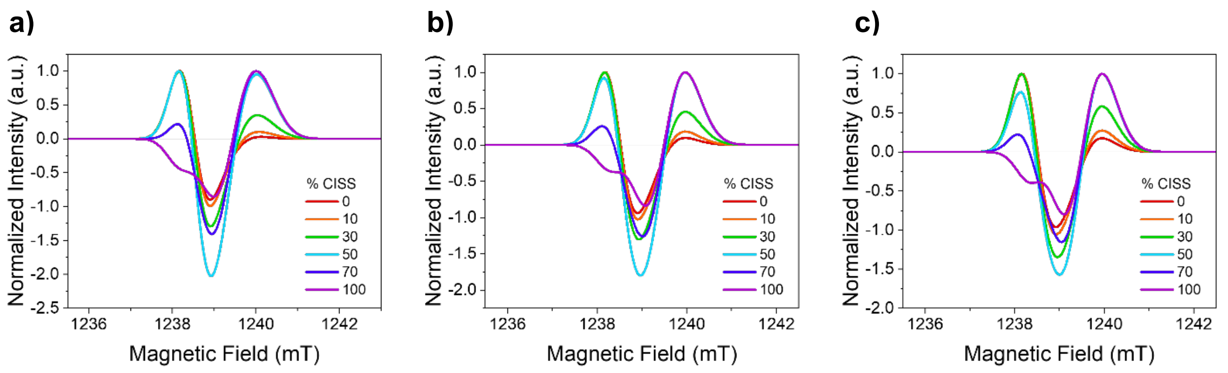

Fig. S10. Q-band TREPR simulations with varying contributions of CISS for (a) **1G** (b) **2G** and (c) **3G.** Simulation parameters are given in Table S4.

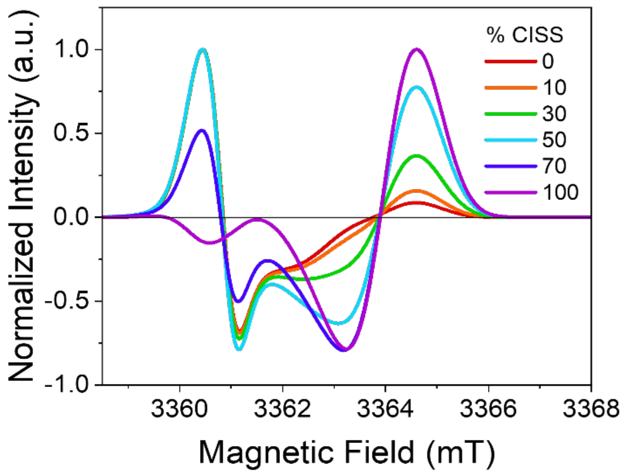

Fig. S11. W-band TREPR simulation with varying contributions of CISS for **1G**. Simulation parameters are given in Table S4.

Additional X-Band Data Analysis

Quality of the TREPR spectral simulations. To demonstrate the quality of the simulations, we show below a fit of the X-band spectrum with higher CISS efficiency of 50% (Fig. S12). Clearly, the smaller intensity of the low-field peak for the singlet compared to the polarized case implies that to reproduce the observed positive peak we need a CISS contribution smaller than that from the singlet, of about 30%.

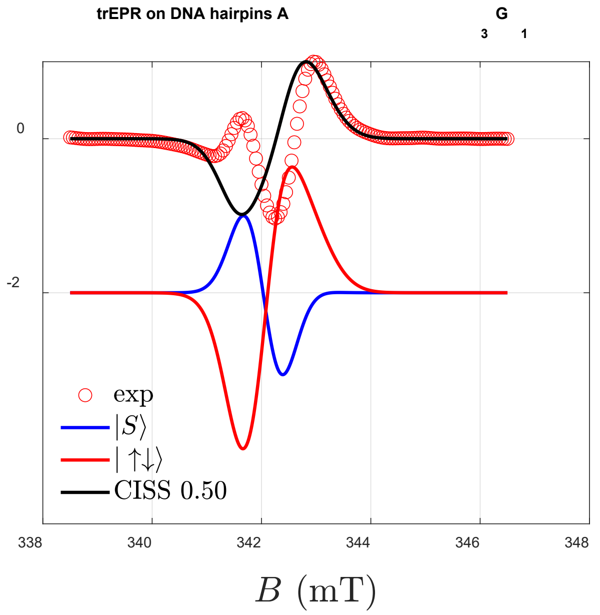

Fig. S12. X-band TREPR of **D-1G** in 50% glycerol/50% buffer (100 mM NaCl and 10 mM Na_3PO_4) 85 K, 150 ns after a 355 nm, 7 ns laser pulse (red circles). The smooth curves overlaying the experimental spectra are simulations using the parameters given in Table S4 with CISS contributions of 0% (blue curve), 100% (red curve), or the 50% CISS (black curve).

Potential effect of radical pair intersystem crossing in $NDI^{\bullet-}$ - $G^{\bullet+}$ on the observed spin polarization of $NDI^{\bullet-}$ - $Sd^{\bullet+}$. Here, we consider the possibility of coherent singlet-triplet mixing occurring in the intermediate $NDI^{\bullet-}$ - $G^{\bullet+}$ before the formation of the final $NDI^{\bullet-}$ - $Sd^{\bullet+}$ state.[3] Using the model of Hore,[3] we compute an illusory "CISS-like contribution" arising from this mechanism, i.e., twice the square of the triplet component in the final radical pair ($p = 2\sin^2\frac{\chi}{2}$ in Eq. (1) of the main text). The results are shown in Fig. S13 using *g*-tensors and hyperfine couplings reported in the figure caption. Different curves refer to different nuclear spin states, while colors denote different molecular orientations. The values fitted from experimental spectra are reported as green circles for comparison. The effect is clearly very small, with values of $p < 0.01$ for all the considered orientations and nuclear spin states. Hence, it cannot explain the additional triplet character attributed to CISS and its magnetic field dependence. In particular, this mechanism only

yields a population of the $|T_0(0)\rangle$ state and not of $|T_{+1}\rangle$ and $|T_{-1}\rangle$, which instead strongly contribute to the observed spectra for a generic orientation of the molecule.

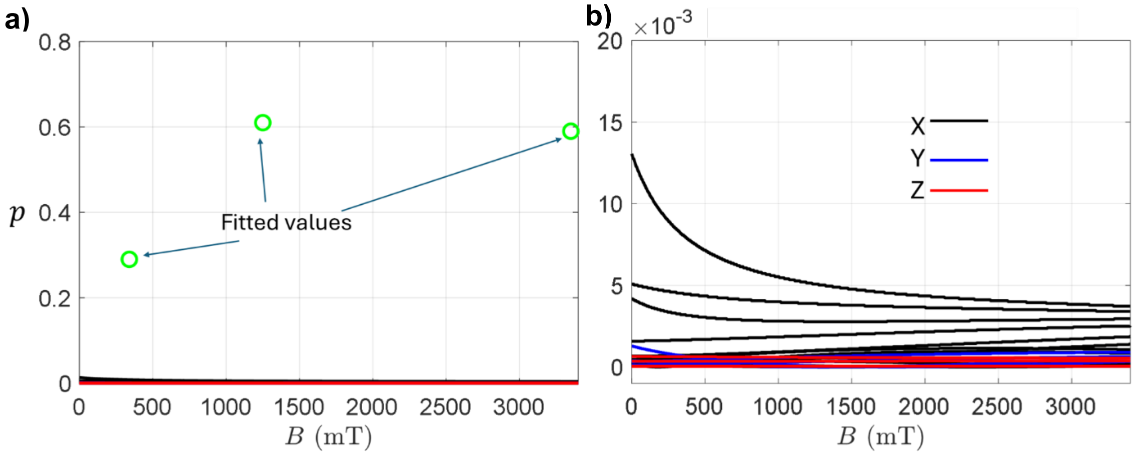

Fig. S13. Effective CISS contribution using a charge transfer rate from the intermediate to the final radical pair of $k = 0.8\,\text{ns}^{-1}$, $g_G = [2.0052, 20033, 2.0022]$, I = 1/2 with [21 29 10] MHz hyperfine coupling and an I = 1 with [36 0 0] MHz coupling. The isotropic exchange in the different steps was neglected, while dipole-dipole interaction was computed assuming a distance of 1 nm in the intermediate state (*D* = 52 MHz). Results for other lengths are analogous. Different colors are for different molecular orientations (black, X, blue, Y, red, Z). a) Comparison with CISS contribution extracted from the TREPR data. B) Expanded view with different curves computed for different nuclear spin states.

Relaxation Theory and Magnetic Field Dependence

A possible example of a simple mechanism bringing eigenstates into play involves relaxation. While radical pair spin relaxation is much slower than EPR measurements, the chiral bridge, potentially excited during the charge transfer, could relax on a much shorter timescale, because it is characterized by much larger energy gaps. Hence, the initial state probed by EPR would be that *after* this relaxation involving the bridge degrees of freedom. Note that this process would then influence the initial state of the SCRP in presence of a coupling between donor/acceptor and the bridge. Given the large energy gaps between the bridge states, this relaxation is likely not affected by B, yielding a SCRP density matrix independent of B in the eigenstate basis. As a result, the initial state inherits a dependence on B because the eigenstates composition depends on B. In

particular, increasing the field and hence $\Delta g \mu_B B$ will yield eigenstates of the SCRP characterized by a larger local spin polarization (more factorized eigenstates).

Note that the above considerations depend only on the form of the eigenstates of the SCRP and on the independence of charge transfer and bridge relaxation on *B,* which are the same if we consider electron (as in model (5)) or hole transfer (as in the present case).

After electron transfer creates the SCRP, the donor-bridge-acceptor system undergoes thermal relaxation to the ground singlet state of the bridge and to a specific mixture of SCRP eigenstates. We focus on relaxation of the bridge charge eigenstates and on that of the combined bridge-donor/acceptor spin eigenstates. These processes are much faster than spin relaxation of the SCRP itself because they involve transitions between states characterized by much larger energy gaps. We evaluate the time evolution of the system density matrix ρ by numerically solving the master equation:[4]

$$\dot{\rho}_{mm'} = \sum_{\gamma\gamma'} R_{mm'\gamma\gamma'} \rho_{\gamma\gamma'}$$

where the Redfield tensor is given by:

$$R_{mm'\gamma\gamma'} = \sum_j \Gamma_j \{ \langle m|H_j|\gamma\rangle\langle\gamma'|H_j^\dagger|m'\rangle \left[D_{m\gamma}\, \delta\left(E_\gamma - E_m - E_{\gamma'} + E_{m'}\right) + D_{m'\gamma'}\, \delta\left(E_{\gamma'} - E_{m'} - E_\gamma + E_m\right)\right] - \delta_{\gamma'm'}\delta\left(E_\gamma - E_m\right) \sum_\alpha \langle m|H_j^\dagger|\alpha\rangle\langle\alpha|H_j|\gamma\rangle] - \delta_{m\gamma}\delta\left(E_{\gamma'} - E_{m'}\right) \sum_\alpha \langle\gamma'|H_j^\dagger|\alpha\rangle\langle\alpha|H_j|m'\rangle \}.$$

Here $|\mu\rangle, |\nu\rangle$ are system eigenstates with energies $E_\mu \neq E_\nu$, H_j are different terms of the Hamiltonian modulated by the coupling to the boson bath and $D_{m\gamma}$ are proportional to the bath spectral function and to the Bose-Einstein factor $n(x) = \left[e^{x/k_B T} - 1\right]^{-1}$, evaluated at the energy gap $|E_\nu - E_\mu|$. To simplify the description and eliminate any dependence on the properties of the vibrational bath, we consider a constant bath spectral density and the low-temperature limit (i.e.,

only transitions which decrease the energy of the system are allowed). Note, however, that transition rates between almost degenerate states ($|E_\nu - E_\mu| \lesssim 100$ MHz) are set to zero. Indeed, these transitions are expected to be very slow. The delta-functions in the expression for $R_{mm'\gamma\gamma'}$ arise from neglecting fast oscillating terms which are averaged out in the relaxation dynamics (secular approximation). This implies that simulations are performed on a coarse-grained time scale, significantly longer than the inverse of the relevant energy gaps in the molecular spectrum.[4]

Here we are focusing on the long-time state after complete relaxation, much slower than the ET dynamics. Hence, to reduce computational effort and study the effect of the applied field, we simulate it starting from the donor-bridge-acceptor density matrix obtained after ET, and for simplicity with an axial spin-orbit coupling within the bridge sites.[5] We then add a typical weak spin-spin dipolar coupling (of strength D) between the two unpaired electrons on the donor and the acceptor $D(S_D^x S_A^x + S_D^y S_A^y - 2S_D^z S_A^z)$ and a Zeeman interaction with an external magnetic field $\mu_B \sum_i \mathbf{B} \cdot \mathbf{g}_i \cdot \mathbf{S}_i$, $\mathbf{g}_i$ being the g-tensor of each site. We have checked that the inclusion of these very small terms has a negligible effect on the ET dynamics before relaxation (characterized by much larger energy gaps). Hence, we can simulate the relaxation dynamics as a function of B and D starting from the charge transferred density matrix obtained without these terms.

The Hamiltonian terms H_j modulated by the coupling to the bath include on-site energies of the bridge orbitals and nearest-neighbor hopping. In addition, we consider the modulation of a isotropic exchange interaction between the electron on the ground state of the donor and that on the first site of the bridge ($H_{D1} = \mathbf{S}_D \cdot \mathbf{S}_1$) and an analogous term between an electron on the last site of the chain and that on the acceptor ($H_{4A} = \mathbf{S}_4 \cdot \mathbf{S}_A$). These terms (not included in the ET dynamics, which is mainly ruled by incoherent transfer steps) ensure complete relaxation of the bridge to its singlet ground state by inducing a polarization transfer from the bridge to D and A.

This model only aims to capture the qualitative trend in the dependence of the spin polarization $p_{DA} = S_A^z - S_D^z$ as a function of the external field. Hence, we simplify the system by assuming isotropic g-tensors with $\Delta g = 0.001$ and typical values of $D = 3$, 5 and 10 MHz, corresponding to donor-acceptor distances of about 2.6, 2.2 and 1.7 nm (in the point dipole approximation).

Our simulations show a final spin polarization p_{DA} monotonically increasing with B. This is due to the more polarized character of the eigenstates as B is increased, while the relaxation process is not significantly affected by B and D. The simulations are shown in Fig. S14 with the external field parallel to the chiral (dipolar) axis (a) and considering an average for $0 \leq \theta \leq \pi$ (b).

We note that the increase in p_{DA} is particularly relevant for the shortest hairpin ($D = 10$ MHz, red trace), where the interplay between D and Δg has the maximum effect on the eigenstates. For

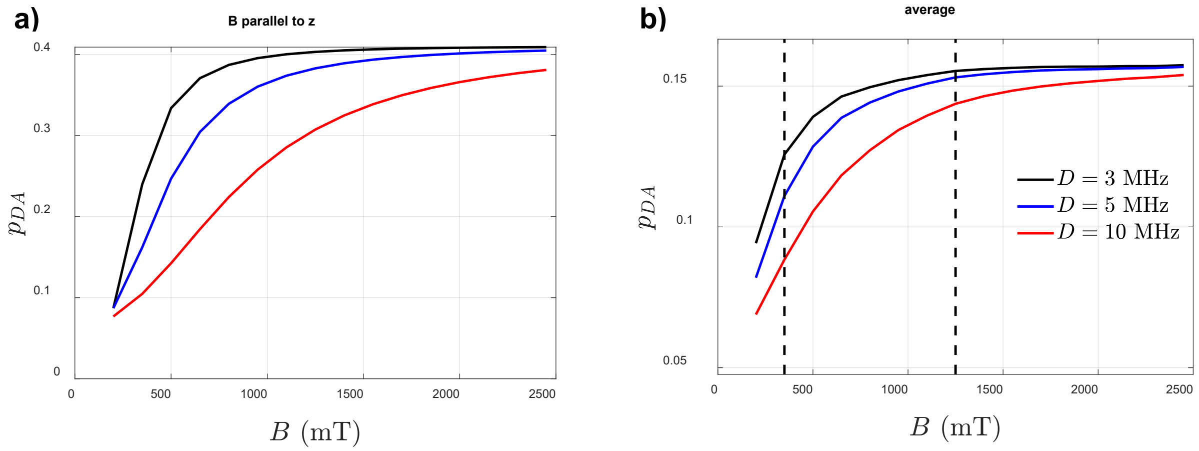

Fig. S14. The simulations are shown a) with the external field parallel to the chiral (dipolar) axis, and b) considering an average for $0 \leq \theta \leq \pi$.

smaller values of D, eigenstates are already almost factorized at $B \sim 1$ T and then polarization saturates. The increase in spin polarization is accompanied by an increase in the triplet character of the initial state probed by TREPR. However, this does not automatically correspond to an increase in the CISS contribution defined in Eq. (1) of the main text when coherences between

eigenstates are lost. Moreover, the calculated increase is mainly in the $|T_0\rangle$ component, while experimental data indicate a remarkable growth of $|T_{+1}\rangle$ and $|T_{-1}\rangle$.

DFT Calculations of EPR Parameters

Geometry optimization calculations of NDI anion and Sd cation were performed using density functional theory (DFT) at B3LYP/def2-TZVP level of theory in Qchem (version 6.1.0).[6] The DFT optimized structures are given below. The *g*-strain tensors of the optimized radical ions were calculated using DFT at B3LYP/EPR-II level of theory using ORCA (version 5.0.4.).[7]

Optimized Geometries for:

NDI anion

C	1.38537443657723	2.43512583976506	-0.00074943431670
C	0.00741415954572	2.43605610706103	-0.00051350525877
C	-0.71324115413146	1.22752414740905	-0.00041250859606
C	-0.02140288247300	-0.00553512083383	-0.00054292969016
C	1.41094760692453	-0.00650155285546	-0.00073834274893
C	2.10311766139167	1.22544292106530	-0.00086138630648
C	-0.71357286756886	-1.23747980991500	-0.00049283450653
C	0.00417065390086	-2.44716267339344	-0.00060640732717
C	1.38213078941532	-2.44809324518035	-0.00076593824640
C	2.10278587891499	-1.23956075185143	-0.00082082982926
C	3.55891950872878	-1.26326698374138	-0.00088395086200
N	4.19889283618005	-0.00953179740876	-0.00120592950733
C	3.55829126937239	1.23895922106343	-0.00120302217847
C	-2.16937471554541	1.25123106706532	-0.00013165125547
N	-2.80934771618308	-0.00250370145256	-0.00012277386654
C	-2.16874640171752	-1.25099516998128	-0.00037287735726
O	4.24101469018744	2.26455942432795	-0.00131616807065
O	4.22396299489719	-2.30024907970083	-0.00138993623413
O	-2.85147051929294	-2.27659485140944	-0.00013875633721
O	-2.83441777084808	2.28821347004330	-0.00008201714591
C	5.65750634559749	0.02087035686504	-0.00160553897846
C	-4.26796140285213	-0.03290624798647	0.00014177520330
H	-4.63157858884707	-0.56480452808540	0.88015501979108
H	-4.63192791969726	-0.56433292314733	-0.88001510236681
H	-4.61911022152451	0.99354606677052	0.00047353041374
H	-0.55602596304124	-3.37266345753983	-0.00056459335534

H	1.94082563125855	-3.37437195288887	-0.00082188951398
H	1.94557162583305	3.36062628347877	-0.00086391142850
H	-0.55128017472481	3.36233505677233	-0.00041133167760
H	6.00865567552987	-1.00558186754577	-0.00146713918724
H	6.02152942650829	0.55273527778291	0.87826017733436
H	6.02106710768393	0.55233047544741	-0.88190979659309

Sd cation

C	4.30315384771793	0.31533014287426	-0.00000123077747
C	4.37019987536145	1.72237716792603	-0.00000123708123
C	3.17531649038023	2.48283888298654	-0.00000376378286
C	1.96072250594692	1.86018131868294	-0.00000193899377
C	1.86189955359034	0.43961216721377	-0.00000181338964
C	3.07379907246321	-0.29810811933857	-0.00000057071275
C	0.63378111915883	-0.27192480036361	-0.00000321222665
C	-0.63378771971525	0.27193439237210	-0.00000306393460
C	-1.86190489293370	-0.43960534108360	-0.00000210555957
C	-3.07380664660747	0.29811089062300	-0.00000042568490
C	-4.30315911715269	-0.31533172291506	0.00000047784964
C	-4.37020022127567	-1.72237856790688	-0.00000197016580
C	-3.17531414836660	-2.48283624858974	-0.00000459445182
C	-1.96072241305170	-1.86017474122710	-0.00000448995939
O	-5.49451460635849	-2.42439784894968	-0.00000150437846
C	-6.77155469055146	-1.76563203066646	0.00000346837540
O	5.49451700127745	2.42439231671305	0.00000019013910
C	6.77155436510670	1.76562101965803	0.00000325390047
H	5.20302505217688	-0.28159109296011	-0.00000019955249
H	3.25806164999521	3.56148368374831	-0.00000333308961
H	1.06596584041179	2.46719646177924	-0.00000470092001
H	3.02488587943994	-1.38026402096364	0.00000005017285
H	0.72774037451328	-1.35209643969399	-0.00000385004604
H	-0.72774808033379	1.35210594918056	-0.00000068752680
H	-3.02489516809530	1.38026698353225	0.00000099713919
H	-5.20303216148329	0.28158658432230	0.00000239870469
H	-3.25805584884918	-3.56148132980840	-0.00000595602855
H	-1.06596437291142	-2.46718827728857	-0.00000647353719
H	-7.50649310572748	-2.56453101138515	0.00000369514218
H	-6.88729920584603	-1.15496236033933	-0.89660024827740
H	-6.88729399300155	-1.15496660698691	0.89661076238474
H	7.50649643151460	2.56451666532766	0.00000298196856
H	6.88729505182591	1.15495134683008	-0.89660097100603
H	6.88729228138035	1.15495458669668	0.89661006530621

References

1. Olshansky, J. H.; Krzyaniak, M. D.; Young, R. M.; Wasielewski, M. R., Photogenerated spin-entangled qubit (radical) pairs in DNA hairpins: Observation of spin delocalization and coherence. *J. Am. Chem. Soc.* **2019,** *141*, 2152-2160.

2. Young, R. M.; Dyar, S. M.; Barnes, J. C.; Juricek, M.; Stoddart, J. F.; Co, D. T.; Wasielewski, M. R., Ultrafast conformational dynamics of electron transfer in exbox^{4+}⊂perylene. *J. Phys. Chem. A* **2013,** *117*, 12438-12448.

3. Hore, P. J., Transfer of spin correlation between radical pairs in the initial steps of photosynthetic energy conversion. *Mol. Phys.* **1996,** *89*, 1195-1202.

4. Tupkary, D.; Dhar, A.; Kulkarni, M.; Purkayastha, A., Fundamental limitations in lindblad descriptions of systems weakly coupled to baths. *Phys. Rev. A.* **2022,** *105*, 032208.

5. Chiesa, A.; Garlatti, E.; Mezzadri, M.; Celada, L.; Sessoli, R.; Wasielewski, M. R.; Bittl, R.; Santini, P.; Carretta, S., Many-body models for chirality-induced spin selectivity in electron transfer. *Nano Lett.* **2024,** *24*, 12133-12139.

6. Shao, Y.; Gan, Z.; Epifanovsky, E.; Gilbert, A. T. B.; Wormit, M.; Kussmann, J.; Lange, A. W.; Behn, A.; Deng, J.; Feng, X.; Ghosh, D.; Goldey, M.; Horn, P. R.; Jacobson, L. D.; Kaliman, I.; Khaliullin, R. Z.; Kuś, T.; Landau, A.; Liu, J.; Proynov, E. I.; Rhee, Y. M.; Richard, R. M.; Rohrdanz, M. A.; Steele, R. P.; Sundstrom, E. J.; Woodcock, H. L.; Zimmerman, P. M.; Zuev, D.; Albrecht, B.; Alguire, E.; Austin, B.; Beran, G. J. O.; Bernard, Y. A.; Berquist, E.; Brandhorst, K.; Bravaya, K. B.; Brown, S. T.; Casanova, D.; Chang, C.-M.; Chen, Y.; Chien, S. H.; Closser, K. D.; Crittenden, D. L.; Diedenhofen, M.; DiStasio, R. A.; Do, H.; Dutoi, A. D.; Edgar, R. G.; Fatehi, S.; Fusti-Molnar, L.; Ghysels, A.; Golubeva-Zadorozhnaya, A.; Gomes, J.; Hanson-Heine, M. W. D.; Harbach, P. H. P.; Hauser, A. W.; Hohenstein, E. G.; Holden, Z. C.; Jagau, T.-C.; Ji, H.; Kaduk,

B.; Khistyaev, K.; Kim, J.; Kim, J.; King, R. A.; Klunzinger, P.; Kosenkov, D.; Kowalczyk, T.; Krauter, C. M.; Lao, K. U.; Laurent, A. D.; Lawler, K. V.; Levchenko, S. V.; Lin, C. Y.; Liu, F.; Livshits, E.; Lochan, R. C.; Luenser, A.; Manohar, P.; Manzer, S. F.; Mao, S.-P.; Mardirossian, N.; Marenich, A. V.; Maurer, S. A.; Mayhall, N. J.; Neuscamman, E.; Oana, C. M.; Olivares-Amaya, R.; O'Neill, D. P.; Parkhill, J. A.; Perrine, T. M.; Peverati, R.; Prociuk, A.; Rehn, D. R.; Rosta, E.; Russ, N. J.; Sharada, S. M.; Sharma, S.; Small, D. W.; Sodt, A.; Stein, T.; Stück, D.; Su, Y.-C.; Thom, A. J. W.; Tsuchimochi, T.; Vanovschi, V.; Vogt, L.; Vydrov, O.; Wang, T.; Watson, M. A.; Wenzel, J.; White, A.; Williams, C. F.; Yang, J.; Yeganeh, S.; Yost, S. R.; You, Z.-Q.; Zhang, I. Y.; Zhang, X.; Zhao, Y.; Brooks, B. R.; Chan, G. K. L.; Chipman, D. M.; Cramer, C. J.; Goddard, W. A.; Gordon, M. S.; Hehre, W. J.; Klamt, A.; Schaefer, H. F.; Schmidt, M. W.; Sherrill, C. D.; Truhlar, D. G.; Warshel, A.; Xu, X.; Aspuru-Guzik, A.; Baer, R.; Bell, A. T.; Besley, N. A.; Chai, J.-D.; Dreuw, A.; Dunietz, B. D.; Furlani, T. R.; Gwaltney, S. R.; Hsu, C.-P.; Jung, Y.; Kong, J.; Lambrecht, D. S.; Liang, W.; Ochsenfeld, C.; Rassolov, V. A.; Slipchenko, L. V.; Subotnik, J. E.; Van Voorhis, T.; Herbert, J. M.; Krylov, A. I.; Gill, P. M. W.; Head-Gordon, M., Advances in molecular quantum chemistry contained in the Q-chem 4 program package. *Mol. Phys.* **2015,** *113*, 184-215.

7. Neese, F.; Wennmohs, F.; Becker, U.; Riplinger, C., The ORCA quantum chemistry program package. *J. Chem. Phys.* **2020,** *152*, 224108.
